# Fragment-based identification of determinants of conformational and spectroscopic change at the ricin active site

**DOI:** 10.1186/1472-6807-7-72

**Published:** 2007-11-06

**Authors:** John H Carra, Colleen A McHugh, Sheila Mulligan, LeeAnn M Machiesky, Alexei S Soares, Charles B Millard

**Affiliations:** 1 United States Army Medical Research Institute of Infectious Diseases, 1425 Porter St., Fort Detrick, MD 21702, USA; 2 The Johns Hopkins Bloomberg School of Public Health, Department of Molecular Microbiology & Immunology, 615 N. Wolfe St., Baltimore, MD 21205, USA; 3 Mount St. Mary's University, 16300 Old Emmitsburg Rd., Emmitsburg, MD 21727, USA; 4 Goldbelt Raven LLC, 10 North Jefferson St., Suite 302, Frederick, MD 21701, USA; 5 Biology Department, 463, Brookhaven National Laboratory, Upton, NY 11973-5000, USA; 6 Walter Reed Army Institute of Research, Division of Biochemistry, 503 Robert Grant Ave., Silver Spring, MD 20910, USA

## Abstract

**Background:**

Ricin is a potent toxin and known bioterrorism threat with no available antidote. The ricin A-chain (RTA) acts enzymatically to cleave a specific adenine base from ribosomal RNA, thereby blocking translation. To understand better the relationship between ligand binding and RTA active site conformational change, we used a fragment-based approach to find a minimal set of bonding interactions able to induce rearrangements in critical side-chain positions.

**Results:**

We found that the smallest ligand stabilizing an open conformer of the RTA active site pocket was an amide group, bound weakly by only a few hydrogen bonds to the protein. Complexes with small amide-containing molecules also revealed a switch in geometry from a parallel towards a splayed arrangement of an arginine-tryptophan cation-pi interaction that was associated with an increase and red-shift in tryptophan fluorescence upon ligand binding. Using the observed fluorescence signal, we determined the thermodynamic changes of adenine binding to the RTA active site, as well as the site-specific binding of urea. Urea binding had a favorable enthalpy change and unfavorable entropy change, with a ΔH of -13 ± 2 kJ/mol and a ΔS of -0.04 ± 0.01 kJ/(K*mol). The side-chain position of residue Tyr80 in a complex with adenine was found not to involve as large an overlap of rings with the purine as previously considered, suggesting a smaller role for aromatic stacking at the RTA active site.

**Conclusion:**

We found that amide ligands can bind weakly but specifically to the ricin active site, producing significant shifts in positions of the critical active site residues Arg180 and Tyr80. These results indicate that fragment-based drug discovery methods are capable of identifying minimal bonding determinants of active-site side-chain rearrangements and the mechanistic origins of spectroscopic shifts. Our results suggest that tryptophan fluorescence provides a sensitive probe for the geometric relationship of arginine-tryptophan pairs, which often have significant roles in protein function. Using the unusual characteristics of the RTA system, we measured the still controversial thermodynamic changes of site-specific urea binding to a protein, results that are relevant to understanding the physical mechanisms of protein denaturation.

## Background

Ricin toxin is a potent ribosome-inactivating protein derived from the castor bean and a relatively common bioterrorism agent [1]. The heterodimeric toxin consists of a lectin B-chain linked by a disulfide bond to a catalytic A-chain (RTA). Free RTA inside the cell can irreversibly inactivate ribosomes by cleaving the glysosidic bond of a specific adenine base in the sarcin/ricin domain of 28S ribosomal RNA [2]. One molecule of this toxin in the cytosol may suffice to kill a human cell [3]. Although death from ricin intoxication can take up to 5 days, no specific therapeutic measures are available for intervention.

Examination of the structural properties and conformational changes of the enzyme's active site is necessary for the discovery of effective inhibitors [4,5]. Induction of a conformational change opening the RTA active site "specificity pocket" has been proposed to be an essential property of an effective inhibitor [5]. We were therefore interested in determining a minimal set of bonding interactions able to stabilize an open conformer appropriate for inhibitor binding, prompting an exploration of very small RTA ligands.

Some purines can function as ricin inhibitors, including adenine, the product of enzymatic cleavage [6]. The structure (1IFS) of recombinant ricin A-chain (RTA) in complex with adenine at the active site [7] revealed that tyrosine 80 had rotated out of its original position to open the catalytic pocket, although the observed electron density for this residue was weak. Stacking of the purine with tyrosine 80 was also observed with other aromatic inhibitors [5,8,9]. We found that a few hydrogen bonds made by an amide group were capable of promoting that conformation; aromatic stacking was not essential. These results suggest that a wider range of molecules, including peptide derivatives, may be explored as components of ricin inhibitors.

We found that the geometry of a cation-pi interaction between the catalytically critical residue arginine 180 and the single tryptophan 211 shifted from parallel towards splayed in response to the presence of small amide-containing ligands. An increase and red-shift in the intrinsic protein fluorescence observed on ligand binding was very similar both to that observed by Watanabe et al. [10] for RTA-adenine, as well as that associated with another arginine-tryptophan interaction in the protein Csk [11]. Fluorescence changes in RTA also provided a useful probe for examination of the specificity and thermodynamics of urea binding, a subject important for understanding the mechanism of protein unfolding [12]. Although urea denaturation is commonly used to determine the free energy changes of protein folding, the physical forces and mechanism underlying this phenomenon are not yet entirely clear.

## Results

### Fluorescence changes induced by active site ligands

**Figure 1:**
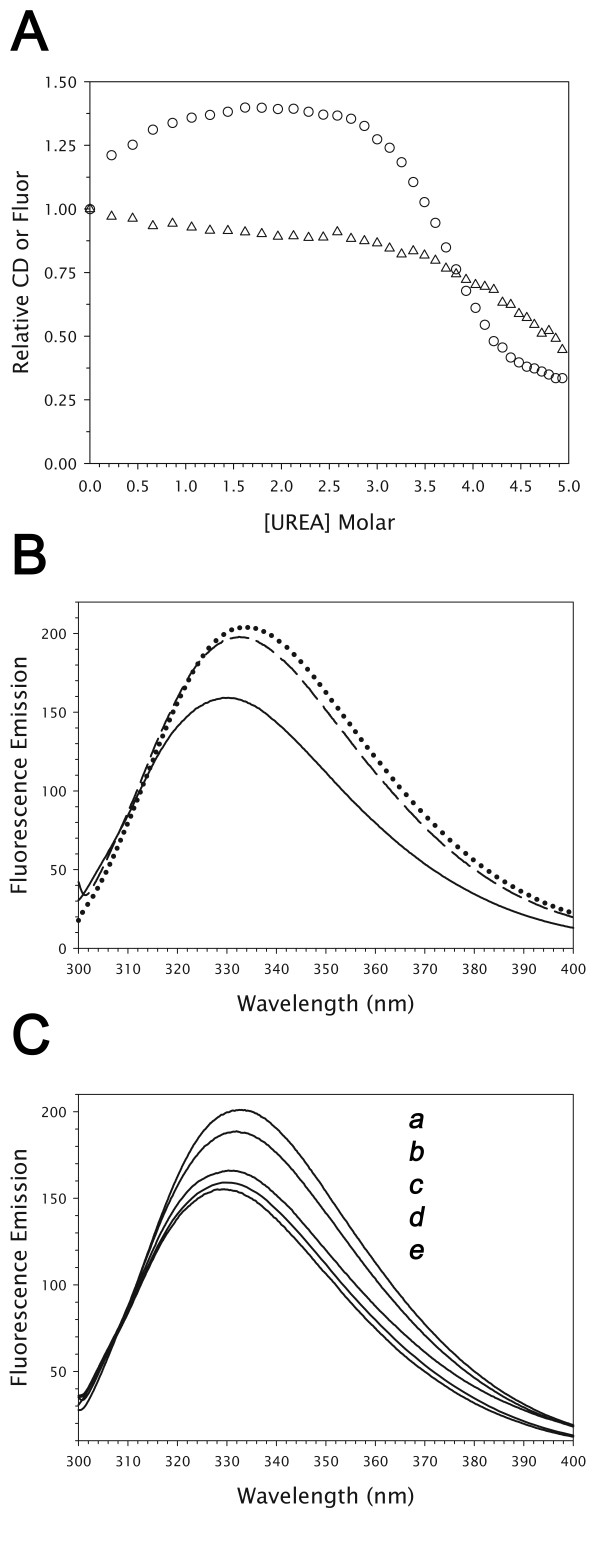
**Compound effects on RTA fluorescence and circular dichroism**. (**A**) Fluorescence emission (circles) and far-UV CD (triangles) changes on titration with urea. (**B**) Fluorescence spectra in PBS buffer alone (solid line), 1 M urea (dashed line) and 2 mM adenine (dotted line). (**C**) Fluorescence in the presence of (*a*) 2 M formamide, (*b*) 1 M N-methylurea, (*c*) 1 M N, N-dimethylurea, (*d*) buffer alone, and (*e*) 1 M hydroxyurea, in order of decreasing maximal intensity.

While studying the unfolding of RTA by urea, we observed a pre-denaturational increase in fluorescence with increasing urea concentration (Fig. 1). The hyperbolic shape of the curve suggested a binding event, therefore we chose to investigate whether urea could bind to RTA at a particular site and influence protein fluorescence. RTA has a single tryptophan, residue 211, located at the active site pocket. Fig. 1 shows emission spectra from RTA in the presence of 2 mM adenine or 1.0 M urea, which have very similar increases in intensity and red-shift from the spectra of RTA alone. The wavelength of maximal emission in both cases increased from 330 to 334 nm. This phenomenon is not caused by urea-induced denaturation, which occurred at higher concentrations as monitored by circular dichroism or fluorescence (Fig. 1). Titration with guanidine-HCl also did not produce a fluorescence increase [13,14].

We next tested a set of small amide-containing molecules related to urea (Fig. 1), and found that at molar concentrations some also produced similar fluorescence increases, including formamide, acetamide and N-methylurea (NMU). In contrast, hydroxyurea, and N, N-dimethylurea did not. A structural explanation for this finding will be provided below.

We hypothesized from these results that urea, formamide, acetamide, and NMU can bind specifically to the RTA active site at the same locus as adenine, also inducing a conformational change resulting in an increase in fluorescence. Inspection of the structures of adenine-bound versus unbound RTA, solved by Weston et al. [7], suggested that a specific H-bond accepted by N3 of adenine from arginine 180 caused this side-chain to rotate away from a co-planar arrangement with tryptophan 211. Such a movement of the charged terminal guanidinium group of arginine could alter the electronic environment of the tryptophan, resulting in the observed fluorescence increase. A similar interaction with arginine 180 might be made either directly or indirectly by the carbonyl group of a urea molecule bound at the same locus as adenine. Unlike urea, guanidine-HCl is capable only of acting as a donor and so could not form this H-bond, consistent with its inability to produce a fluorescence increase.

Decrease of urea concentration by ten-fold dilution from 1.5 M showed that the fluorescence change was reversible, whereas RTA denaturation was largely irreversible (not shown). Near-UV CD spectra (not shown) of RTA in 1.5 M or 0 M urea both had a strong negative peak at 295 nm. This indicated that tryptophan 211 was present in an asymmetric electronic environment in both cases, consistent with preservation of local tertiary structure at the active site. The UV absorbance spectrum of RTA also was not affected by 1.5 M urea. The non-coincidence of denaturational processes observed by circular dichroism versus fluorescence (Fig. 1) is due to the presence of an intermediate state in unfolding of RTA [14].

### RTA-ligand complexes

**Figure 2:**
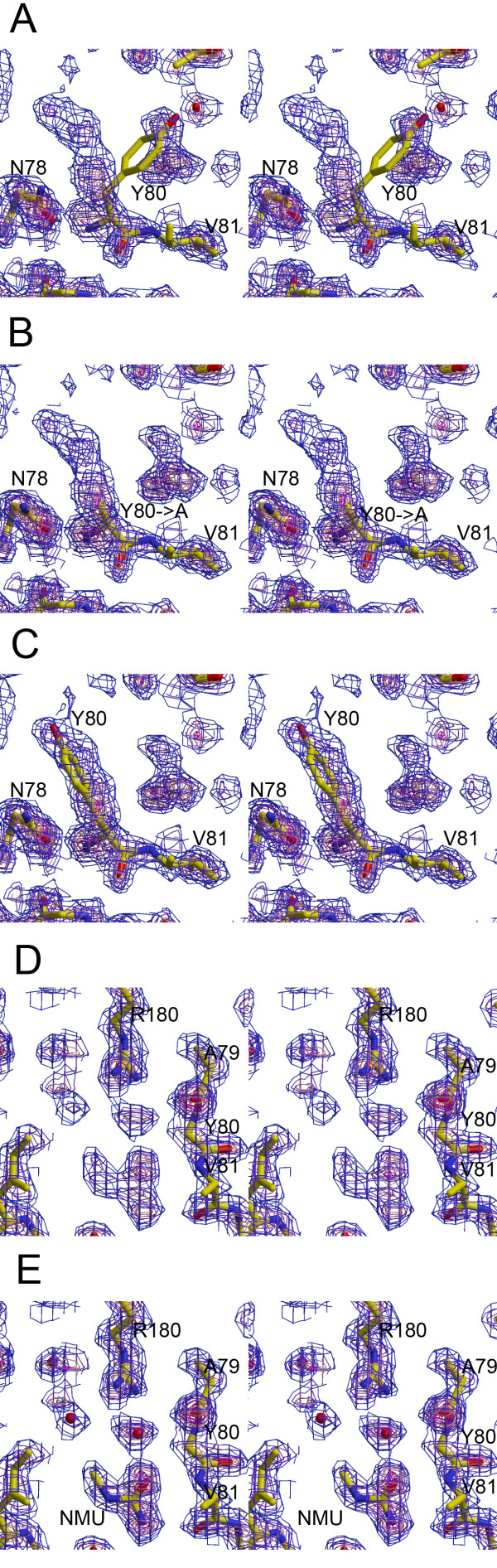
**Electron density maps of the RTA-NMU complex instereo**. (**A**) Initial 2mFo-DFc map before repositioning of Tyr80. Contoured at 1σ (blue), 2σ (purple), 3σ (pink), 4σ (magenta), and 5σ (red). (**B**) Density recalculated after removal of the Tyr80 ring. (**C**) After repositioning Tyr80 to fit the observed density and one round of refinement. (**D**) Density before placement of N-methylurea and water molecules at the active site. (**E**) Final structure after placement of ligand and water.

To test for ligand binding, we soaked RTA crystals in solutions of mother liquor containing 2 M NMU, 1 M urea, or 2 M acetamide. These concentrations were chosen to maximize ligand occupancy while avoiding protein denaturation. The highest quality result was obtained with NMU. This structure was refined to 1.8 Å resolution with an *R*_cryst _of 22.5% and an *R*_free _of 24.3% (Table 1). The electron density for tyrosine 80 obtained by molecular replacement phasing with the RTA structural model 1IFT indicated that this residue had moved in the presence of NMU (Fig. 2). Upon refining tyrosine 80 in an alternate conformation at full occupancy, positive-difference electron density consistent with an NMU molecule at the active site became clear in 2mFo-DFc maps. We refined an NMU molecule in this density as shown in Figure 2, along with a proximal water molecule. The asymmetry and hydrogen-bonding potential of NMU supported placement of the ligand with a unique orientation.

**Table 1:** X-ray data collection and refinement statistics.

**Data Collection**	**RTA – NMU**	**RTA – urea**	**RTA – acetamide**	**RTA- adenine**
Space Group	P 4_1_2_1_2	P 4_1_2_1_2	P 4_1_2_1_2	P 4_1_2_1_2
Unit cell dimensions (Å)				
*A*	67.73	67.37	67.11	68.16
*B*	67.73	67.37	67.11	68.16
*C*	140.77	141.14	141.32	141.20
Resolution range (Å)^*a*^	30–1.80 *(1.91–1.80)*	23–2.40 *(2.55-2.40)*	34–2.09 *(2.22-2.09)*	34–1.94 *(2.06-1.94*)
*R*_sym_^*a*^	0.064 (*0.455)*	0.114 *(0.373)*	0.093 *(0.350)*	0.128 *(0.339)*
Completeness (%) ^*a*^	99.6 (*99.9)*	99.7 *(99.8)*	99.9 *(99.8)*	99.9 *(99.9)*
Redundancy	7.0	4.4	17.9	12.4
Unique reflections	31,106	13,295	19,857	26,260
*I*/σ*I*^*a*^	34.0 *(4.80)*	*8.9 (3.7)*	7.09 *(1.71)*	17.8 *(8.88)*
**Refinement**				
Total atoms	2,178	2,118	2,157	2,197
Protein atoms	2,038	2,038	2,037	2,037
Water atoms	125	66	106	134
Ligand atoms	5	4	4	10
Sulfate atoms	10	10	10	15
*R*_cryst_^*b*^	0.231 *(0.252)*	0.252 *(0.286)*	0.234 *(0.252)*	0.265 *(0.270)*
*R*_free_^*b*^	0.247 *(0.267)*	0.297 *(0.355)*	0.272 *(0.319)*	0.301 *(0.304)*
R.m.s. deviations				
Bond length (Å)	0.0050	0.0066	0.0075	0.011
Bond angle (°)	1.10	1.16	1.25	1.20
Mean temperature factor (Å^2^)				
For all atoms	22	24	21	20
For ligand atoms	20	17	21	21

^*a *^Values in parentheses are for the highest resolution bin. ^*b *^*R*_cryst _= Σ|_o _– F_c_|/Σ|F_o_| for reflections in the working set, as calculated by CNS; *R*_free _is for a 5% test set of reflections excluded from crystallographic refinement.

**Figure 3:**
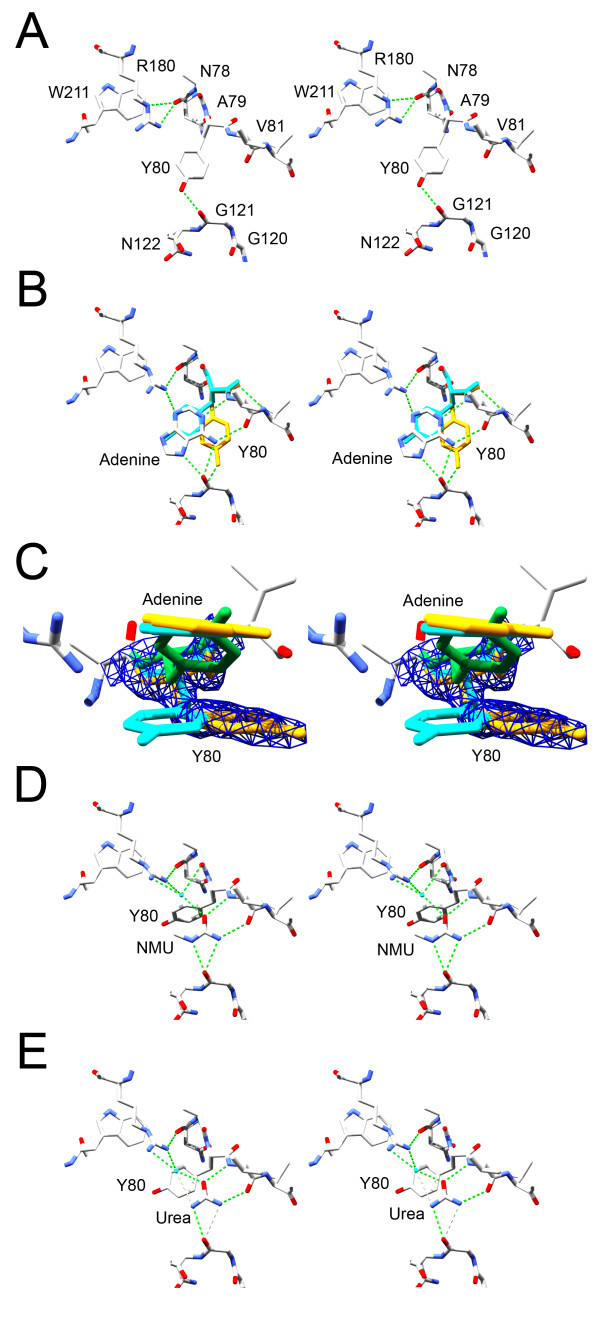
**Ligand effects on active site conformation**. (**A**) RTA without bound ligand (1IFT [7]). Figure made in stereo with SPDBV [46] and POV-Ray. (**B**) RTA with bound adenine. The position of Tyr80 in structure 1IFS is shown in cyan, and in 2P8N in yellow. (**C**) Closer view of the effect of binding adenine on the active site. Tyr80 in structure 1IFT (without adenine) is in green, 1IFS in cyan, and 2P8N in yellow. The 2mFo-DFc electron density of Tyr80 in 2P8N is contoured at 1.5σ. (**D**) RTA-NMU complex. A single water molecule is shown in light blue. (**E**) RTA-urea complex.

As in the RTA-adenine complex 1IFS [7], arginine 180 rotated out of plane with the stationary indole ring of tryptophan 211 in the presence of NMU (Fig. 3), and to a greater angle. In the complex with NMU, a single ligand molecule was observed occupying the same position as the adenine base, lying nearly in the same plane. No other molecule of NMU was found. A water molecule (number 384) was positioned to donate an H-bond to the carbonyl group of NMU, while simultaneously forming bidentate H-bonds with the side-chain of arginine 180. This water was associated with a strong electron density peak and a B-factor of 20.7 Å^2^, suggesting that it was relatively tightly bound. NMU also was positioned to form H-bonds with the peptide backbone at residues valine 81 and glycine 121, comparable with those proposed for the adenine complex. The refined structure of RTA in the N-methylurea complex was overall very similar to that of unbound RTA (1IFT) with a root-mean-square-deviation of 0.84 Å for all protein atoms.

The rotameric position of Tyr80 shifted in the presence of all the studied ligands (Fig. 3), allowing access to potential H-bond partners in the active site side-chains. However, we found that the position of Tyr80 in a newly determined structure of RTA soaked in 2 mM adenine was not the same as that previously reported ([7]). It may be important to note that the 1IFS RTA-adenine complex was prepared differently; pre-formed RTA crystals were soaked with AMP, which apparently resulted in catalytic cleavage of the glycosidic bond to yield an adenine bound in the active site. The position of the Tyr80 in structure 1IFS was relatively disordered, with a calculated occupancy of 0.6 for atoms in the ring and an average B-factor of 42 Å^2 ^versus 27 Å^2 ^for all atoms. In the RTA-adenine complex 2P8N, we observed strong positive difference electron density at the 4σ level in omit maps calculated by excluding this residue, allowing an alternative position to be determined as shown in Fig. 3. The average B-factor of ring carbons for Tyr80 was 30 Å^2 ^versus 21 Å^2 ^in all atoms. The electron densities of the ring and Cα-Cβ-Cγ bonds were clear throughout refinement, indicating re-orientation of the side-chain to the relatively common t80° rotamer [15]. In this position, the hydroxyl group of Tyr80 can hydrogen bond with the amide nitrogen of Gly121. The positions of the adenine ligand and Arg180 in this structure were comparable to those observed in the 1IFS model.

Movement of the charged guanidinium group of Arg180 relative to the tryptophan provides a clear structural explanation for the increase and red-shift in fluorescence emission observed by our group and Watanabe et al. [10]. The angle formed from the tryptophan 211 phenyl ring centroid to NE and HE of arginine 180 changed from 87° in RTA alone (1IFT) to 58° in the adenine complex (1IFS), and 37° in the RTA-NMU complex.

The structures of RTA complexes with urea (Fig. 3) or acetamide (not shown) were also solved. Urea was more disruptive to the crystals than was NMU, but we were able to collect a complete dataset on a crystal soaked in 1 M urea diffracting to 2.4 Å. The angle adopted by the tyrosine 80 ring was more co-planar to the ligand in the presence of urea versus NMU, perhaps reflecting the absence of steric interference with the ring from the methyl group of NMU. A single water molecule was positioned to H-bond with both the urea molecule and the side chain of arginine 180. Acetamide, which lacks the second amino nitrogen of urea and can make H-bonds only from an amide group, yielded a similar structure.

### Binding constants and energetics

**Figure 4:**
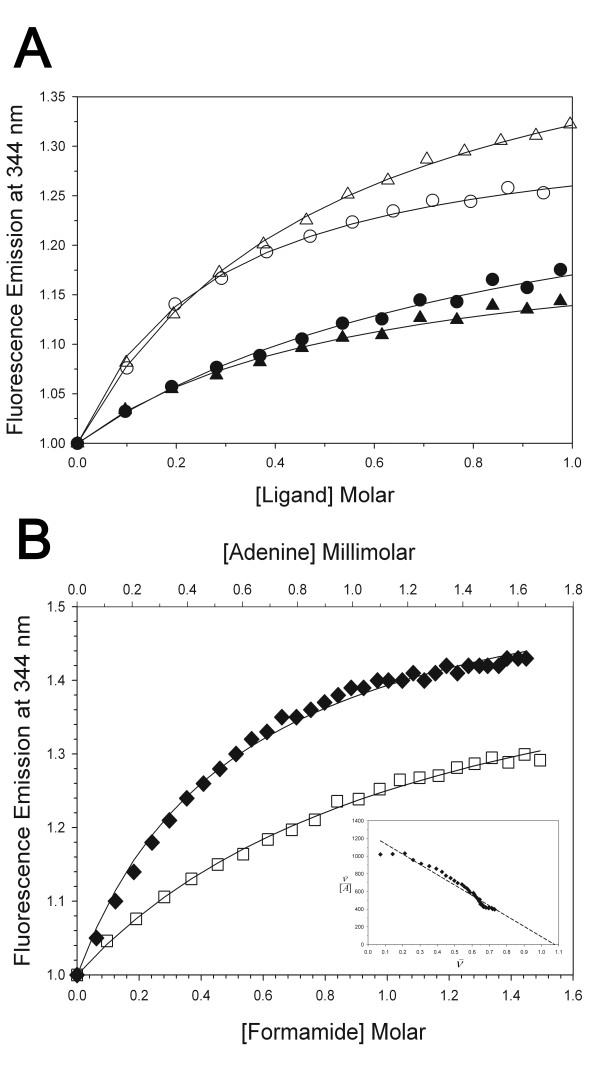
**Binding isotherms**. (**A**) Fluorescence emission increase on titration of RTA with urea at 5°C (open circles) and 30°C (open triangles), and NMU at 5°C (filled circles) and 30°C (filled triangles). (**B**) Titration with adenine (diamonds) and formamide (squares) at 25°C. The axis for adenine is in millimolar (top). A Scatchard plot for adenine binding is inset.

Fluorescence emission provided a useful probe to determine binding constants and thermodynamic changes for the compounds tested, including adenine (Table 2). Titration of the ligands onto RTA as monitored by fluorescence intensity at 344 nm yielded hyperbolic curves (Fig. 4) that could be well described by non-linear regression with a model of single-site binding. The K_D _of urea for RTA at 25°C was 0.5 M (Table 2). Its affinity was temperature-dependent, being stronger at lower temperature due to a negative enthalpy change of binding. Formamide and acetamide bound more weakly than urea, with K_D _values of 0.9 and 1.4 M respectively, presumably due to their lack of a second amino H-bond donor. A greater maximal fluorescence emission increase was observed at higher temperatures, but this may be due more to the intrinsic temperature dependence of indole fluorescence rather than differences in molecular interactions with the protein.

**Table 2:** Energetics of ligands binding to RTA.

Compound	K_D _(M)^*a*^	ΔG° (kJ/mol)^*a*^	ΔH° (kJ/mol)	ΔS° (kJ/(K*mol))
Urea	0.5 ± 0.09	-2 ± 0.6	-13 ± 2	-0.04 ± 0.01
N-methylurea	0.9 ± 0.04	-0.2 ± 0.09	---	---
Acetamide	1.4 ± 0.03	0.8 ± 0.06	---	---
Formamide	0.9 ± 0.1	0 ± 0.3	---	---
Adenine	(7 ± 0.5) × 10^-4^	-20 ± 0.6	-53 ± 3	-0.12 ± 0.01

^a ^Results are the average and standard error of three measurements.

In contrast, hydroxyurea at 1 M gave no detectable fluorescence increase (Fig 1c). The difference in behavior of NMU versus hydroxyurea is explicable with reference to the crystal structures. Modeling hydroxyurea onto the NMU complex places it into an unfavorable environment without adequate H-bonding partners for the hydroxyl group. N, N-dimethylurea appeared to bind weakly if at all in fluorescence experiments (Fig. 1), and a dissociation constant could not be determined in that case. Steric interference with phenylalanine 93 by the second methyl group of the ligand likely prevented binding in that case.

Watanabe et al. [10] found that binding of adenine to the RTA depurination site as monitored by fluorescence had a K_D _of 1 mM, in agreement with our figure of 0.7 ± 0.05 mM (Table 2). Those authors found a non-linear Scatchard plot for adenine binding, suggesting the presence of two sites of differing affinity. However, our data were well described by a model of a single site (Fig. 4), using either non-linear regression or Scatchard analysis (R = 0.98). A ligand stoichiometry of one was justified by the crystallographic results showing one molecule of each ligand at the active site. While it is possible that other ligand molecules associated with the protein at other loci, no suitable electron density for other bound ligands was observed. Moreover, only the ligand bound as shown in Figure 3 has a clear mechanism to influence the environment of the sole indole ring fluorophore, by altering the position of arginine 180. Ligand molecules in other areas of the protein would be spectroscopically silent if present.

**Figure 5:**
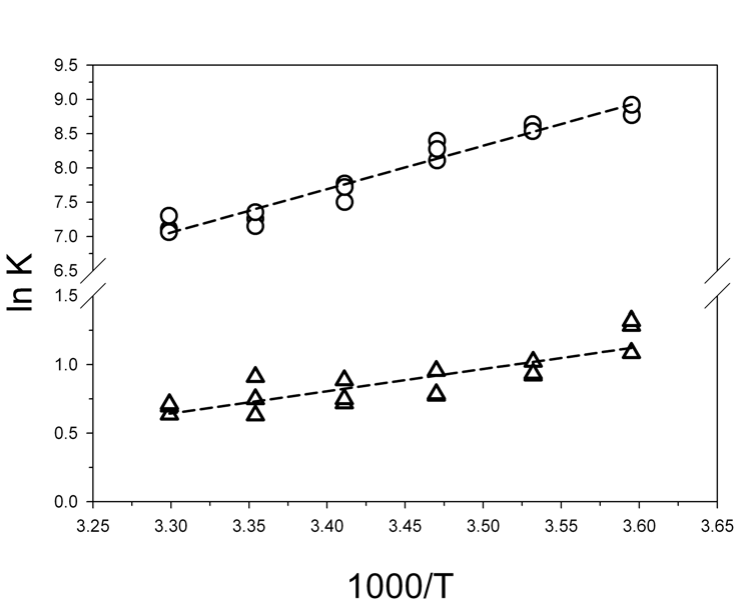
**van't Hoff plots**. Temperature dependence of binding of urea (triangles) or adenine (circles) to RTA.

To find the enthalpy and entropy changes associated with binding of urea or adenine to RTA, titration experiments were carried out over a temperature range from 5 to 30°C (Fig. 5). The temperature range was limited to avoid effects from irreversible unfolding of the protein. Van't Hoff analysis gave a ΔH° for urea binding of -13 ± 2 kJ/mol. The amount of scatter in the data was too large and the temperature range too narrow to determine ΔC_p _by fitting with a temperature-dependent enthalpy change. However, the slight deviation from linearity of this plot (R = 0.85) indicated that the ΔC_p _of binding must be relatively small. An unfavorable entropy change of -0.04 ± 0.01 kJ/(K*mol) opposed the enthalpy change, resulting in a ΔG° of -2 ± 0.6 kJ/mol. For the larger and tighter-binding ligand adenine, ΔH° was -53 kJ/mol (R = 0.97) with a ΔS° of -0.12 ± 0.01 kJ/(K*mol). NMU, acetamide, and formamide gave complex non-linear results in van't Hoff analysis that did not allow determination of ΔH and ΔS, perhaps due to their lower affinities for the protein.

## Discussion

### Effects of changes in geometry of Arg180-Trp211

**Figure 6:**
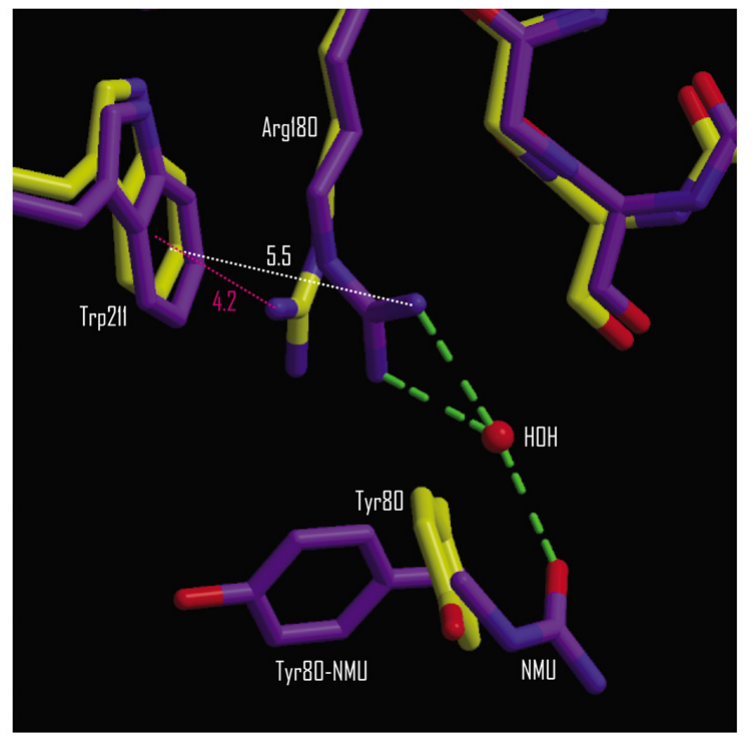
**Geometry of the arginine 180 – tryptophan 211 interaction**. The structure of RTA without a ligand (1IFT [7]) is in yellow, overlaid on the RTA-NMU complex in purple. Distances from the ring centroid to arginine 180 NH1 in the RTA-NMU complex are indicated.

Arginine 180 of RTA plays a crucial role in catalysis, probably by protonation of the adenine leaving group [8,16,17]. In the structure of RTA complexed with NMU (Fig. 2 and 6), the ligand apparently made an H-bond to a water molecule which was linked through bidentate H-bonding to arginine 180. As a result, the relationship of the arginine and tryptophan residues changed from a "stacked" geometry towards a "splayed" one [18]. The distance of the NH1 atom of arginine 180 to the centroid of the tryptophan 211 aromatic ring increased from 4.2 Å in RTA alone to 5.5 Å in the RTA-NMU complex. By comparison, in the RTA-adenine structure of Weston et al. this distance was 5.1 Å.

The intensity increase and red-shift in fluorescence emission observed when Arg180 rotates away from Trp211 could be attributed to a release from quenching, as was proposed by Watanabe et al. [10]. However, arginine is not known to be a quencher of indole fluorescence [19]. Alternatively, specific interactions made to the tryptophan by arginine in the splayed geometry could be responsible for an enhancement of fluorescence. A similar difference in fluorescence emission has been observed with mutants of the tyrosine kinase Csk. In the catalytic domain of Csk, Arg318 makes a cation-pi interaction to Trp352, with the terminal guanidinium perpendicular to the face of the indole ring [20]. Using an alanine substitution of the arginine, Lee et al. [11] showed that the presence of this interaction was responsible for an approximately 35% increase and 3 nm red-shift in tryptophan fluorescence, very close to the difference in emission observed between the two conformers of RTA. A red-shift in fluorescence emission is normally attributed to an increase in environmental polarity experienced by the tryptophan, often due to greater solvation. However, in the case of RTA, the local electronic environment and λ_max _appear to be significantly affected by the proximity and angular relationship of the indole ring to a charged side-chain.

Cation-pi interactions are known to participate in substrate binding and enzyme catalysis [21], as well as key structural interactions within and between protein subunits [22,23]. Important functional roles are often played by arginine residues paired with aromatics [24-26], fifty percent of which are coplanar [22]. A correlation of fluorescence spectral changes with shifts in this residue-pairing geometry, if generalizable, may provide a useful tool to examine structure-function relationships in other proteins.

The Arg180-Trp211 cation-pi interaction also appears to be important for stable folding of RTA. Substitution of arginine 180 with neutral amino acids yielded unstable proteins that precipitated during expression [16], while R180H was stable only when the histidine was positively charged at less than neutral pH [27]. Histidine replacement of Arg180 decreased k_cat _by greater than a thousand-fold. Replacement with lysine (R180K) reduced activity only four times, consistent with a role for this residue in donation of a proton to N3 of adenine [16,17].

Tyrosine 80 also changes position on ligand binding and may be able to affect Trp211 fluorescence. However, we find this less plausible because Tyr80 is relatively distant from Trp211 compared to Arg180. The distance in the RTA-adenine complex 2P8N from Trp211 CZ3 to Tyr80 CD2 was 7.5 Å, versus 3.9 Å to Arg180 CZ. The RTA-urea and RTA-adenine complexes also showed similar fluorescence spectra (Fig. 1) and a splayed arrangement of Arg180, while the positions of Tyr80 in these complexes differed (Fig. 3). The orientation of Tyr80 did not therefore appear to control the fluorescence emission of Trp211.

### Thermodynamic changes of urea binding

The fortuitous existence in RTA of a conformational switch triggered by ligand binding provided a convenient signal for monitoring the binding of urea and other ligands. We were thereby able to determine for the first time the thermodynamic changes associated with binding of a single urea molecule to a protein. Although the binding observed was weak, it was specific to the depurination locus, as evidenced by the RTA-urea complex structure. The energies of urea-protein interactions are relevant to understanding of the mechanism of denaturation by urea; a fundamental issue in protein folding that remains under discussion. Models proposed for this mechanism include those based on specific binding of urea molecules, indirect effects on solvent structure and protein hydrophobicity, and competition for H-bonding with the protein backbone [28]. Because urea is a commonly used chemical scaffold, drug design approaches that utilize thermodynamic information for ligand optimization may also benefit from basic data on the enthalpy and entropy changes of urea binding [29].

In this analysis, we employed a simple binding isotherm instead of the solvent exchange model of Schellman [30] often used for the analysis of preferential hydration when denaturant molecules and water compete for a weak binding site on a protein [31]. On the basis of the determined structures, the binding of amide ligands to the RTA active site is to a locus previously occupied by a protein side-chain (Fig. 2), not water, therefore the use of a solvent exchange model did not appear to be justified in this case.

The value of -13 ± 2 kJ/mol we obtained for the ΔH° of urea binding by van't Hoff analysis can be compared to that of Makhatadze and Privalov [32], who arrived at a figure of -9 kJ/mol for general protein-urea interactions using a combination of flow-mixing and scanning calorimetry. In contrast, Zou et al. [33] gave a value of -34 kJ/mol for urea-amide interactions obtained by dissolution of cyclic dipeptides into urea solutions. In comparing these values, it should be kept in mind that the ΔH° we measured for urea binding to RTA was for one specific site, not an average over many different interactions. It also necessarily includes the enthalpy change associated with the shift of side-chain positions at the active site, although this is likely to be small. The extent to which these thermodynamic results for urea binding can be generalized to other loci is as yet unclear, due to a paucity of other data on site-specific binding. However, Gill et al. [34] reported a ΔH° value of -14.2 ± 2.1 kJ/mol for urea binding to the peptide model diketopiperazine. This is the same as our figure within error, suggesting that the ΔH° value we have determined is consistent with urea/peptide interactions in general.

An increase in fluorescence of RNase T1 in the early phase of a guanidine-HCl denaturation experiment has also been observed [35] and attributed to specific binding of a guanidine cation to the folded state. As Pace et al. concluded, adequately strong and specific binding of a denaturant molecule can significantly affect the shape of a denaturation transition curve, producing non-linearity in plots of ΔG as a function of denaturant concentration and discrepancies in *m *values. The irreversibility of RTA unfolding precludes that analysis in this case. Few other examples exist in the literature of discrete protein binding sites identified for urea. This is at least partly due to the difficulty of measurement and detection of interactions with very weak affinities. Urea at 8 M concentration was observed to bind to lysozyme at nine loci, including the "acetamido-specific" C subsite [36,37]. However, the binding constants for these interactions are not known. At 8 M urea, solvent reorganization results in a weakening of the hydrophobic effect [12]. However, up to 1 M urea, changes in solvent structure as predicted by molecular dynamics are small [28], allowing the thermodynamic changes we measured to be attributed directly to binding of urea to the RTA active site.

### Implications for RTA inhibitor design

Investigation of small-molecule inhibitors of ricin has so far mainly focused on nucleotide and RNA substrate analogs [38]. The strongest inhibitors reported in the literature to date are nanomolar-range K_i _molecules incorporating a pyrrolidine transition-state analog moiety into an RNA stem-loop structure [39]. Miller et al. [5] proposed that opening of the RTA active site's "specificity pocket" by displacement of the tyrosine 80 ring is an important characteristic for a good inhibitor. Identification of the necessary bonding interactions promoting this shift is therefore significant for the discovery of therapeutic compounds. Earlier structures of complexes of RTA with adenine and other inhibitory compounds including formycin monophosphate and pteroic acid revealed their aromatic rings stacking with tyrosine 80 in its "open" or displaced position [7-9]. Upon examination of a crystal complex of RTA with adenine formed by soaking with adenine instead of AMP, we found that the overlap of the tyrosine 80 ring with the purine was much less than previously reported, due to a difference in side-chain rotameric position.

The alternative orientation of Tyr80 in the adenine-bound complex 2P8N compared with that observed in 1IFS suggests that the contribution of stacking to ligand binding is small, or that offset stacking is more favorable than direct overlap. Therefore it may be possible to identify other classes of inhibitors that do not rely on aromaticity to bind this pocket, although purines and their derivatives clearly have the greatest affinity of compounds examined to date. A possible H-bond of the Tyr80 hydroxyl with the Gly121 backbone may also favor the position shown in 2P8N. Displacement of Tyr80 was also observed in the RTA-NMU and RTA-urea complex structures (Fig. 3), although the resulting side-chain position was not identical. The apparent difference in Tyr80 Chi2 angles between the RTA-NMU and RTA-urea complexes may reflect steric constraints imposed by the methyl group of NMU.

## Conclusion

Using a "fragment-based" approach [40] combining spectroscopic and crystal structure data on the ricin A-chain, we identified a minimal set of ligand interactions able to produce positional shifts of the critical active-site residues arginine 180 and tyrosine 80. Hydrogen bonds made by an amide group alone were sufficient to produce a conformational change at the active site pocket, while aromatic stacking of the ligand with tyrosine 80 was not absolutely necessary. The range of useful compounds for construction of ricin inhibitors may, therefore, be larger than previously anticipated. We also propose a plausible mechanistic explanation for the origin of a shift in tryptophan fluorescence emission involving a change in geometry of a cation-pi interaction. Using the spectral characteristics of the RTA system, we measured the thermodynamic changes of site-specific binding of urea to a protein, results that bear on models of the mechanism of urea-induced denaturation.

## Methods

### Spectroscopic experiments

RTA was expressed in *E. coli *and purified by ion exchange chromatography. Circular dichroism and dynamic light scattering measurements showed that the protein was an appropriately folded, monodisperse monomer. Titration experiments were carried out using a Peltier-controlled Jasco J-810 spectropolarimeter with a fluorescence attachment. PBS buffer at pH 7.4 was used, except in the case of adenine, where it was necessary to use 100 mM Na-Phosphate, pH 7.4, 0.1 mM dithiothreitol to prevent pH shifts and protein oxidation upon adenine addition. Solutions were made within 1 day of use, and formamide was thoroughly deionized with AG501-X8 resin (Bio-Rad). Concentrated ligand solutions were titrated into 0.1 mg/mL protein solutions while tryptophan fluorescence was excited at 295 nm and emission monitored. Emission at 344 nm (3 nm bandwidth) was chosen to maximize the observable signal change. Isotherms were fit to the equation:

θ=K[L]1+K[L]
 MathType@MTEF@5@5@+=feaafiart1ev1aaatCvAUfKttLearuWrP9MDH5MBPbIqV92AaeXatLxBI9gBaebbnrfifHhDYfgasaacPC6xNi=xI8qiVKYPFjYdHaVhbbf9v8qqaqFr0xc9vqFj0dXdbba91qpepeI8k8fiI+fsY=rqGqVepae9pg0db9vqaiVgFr0xfr=xfr=xc9adbaqaaeGacaGaaiaabeqaaeqabiWaaaGcbaacciGae8hUdeNaeyypa0tcfa4aaSaaaeaacqWGlbWscqGGBbWwcqqGmbatcqGGDbqxaeaacqaIXaqmcqGHRaWkcqWGlbWscqGGBbWwcqqGmbatcqGGDbqxaaaaaa@3AD0@

where Θ is the fractional saturation with ligand, *K *is the binding constant, and [L] is the ligand concentration. For urea and NMU, data to 1 M concentration were used in fitting to avoid effects associated with irreversible denaturation at higher concentrations. For the non-denaturing compounds acetamide and formamide, data to 1.5 M were used. Temperature was varied over the range of 5°C to 30°C to determine the temperature dependence of *K*. ΔH° and ΔS° were then found using the van't Hoff equation:

ln⁡K=−ΔH°RT+ΔS°R
 MathType@MTEF@5@5@+=feaafiart1ev1aaatCvAUfKttLearuWrP9MDH5MBPbIqV92AaeXatLxBI9gBaebbnrfifHhDYfgasaacPC6xNi=xI8qiVKYPFjYdHaVhbbf9v8qqaqFr0xc9vqFj0dXdbba91qpepeI8k8fiI+fsY=rqGqVepae9pg0db9vqaiVgFr0xfr=xfr=xc9adbaqaaeGacaGaaiaabeqaaeqabiWaaaGcbaGagiiBaWMaeiOBa4Maem4saSKaeyypa0tcfa4aaSaaaeaacqGHsislcqqHuoarcqqGibascqGHWcaSaeaacqqGsbGucqqGubavaaGaey4kaSYaaSaaaeaacqqHuoarcqqGtbWucqGHWcaSaeaacqqGsbGuaaaaaa@3FF6@

### Crystallization and X-ray data collection

Tetragonal crystals of RTA were obtained at 22°C by vapor diffusion in hanging drops [7]. Crystals were harvested and soaked in artificial mother liquor (1.8 M ammonium sulfate, 50 mM sodium acetate, pH 4.2) containing the ligand molecule for 48 hours before transfer to paratone-N oil and flash-cooling in liquid nitrogen. Ligand concentrations used were 2 M NMU, 1 M urea, and 2 M acetamide. For the acetamide-RTA structure, data were collected at 100 K on a Bruker FR591 high flux, rotating anode X-ray diffractometer (PROTEUM) with a SMART 6000 2K CCD detector at WRAIR. For the adenine, NMU and urea complexes, data were collected at 100 K on beamlines X29 and X12C at the National Synchrotron Light Source, Brookhaven National Laboratory.

Structures were solved by molecular replacement in space group P4_1_2_1_2 using AMoRe (CCP4) with the coordinates of PDB entry 1IFT as a search model. Initial inappropriate placement of the side chain of Tyr80 was evident in m*F*_o _– *DF*_c _difference maps created with XtalView [41]. The Tyr80 ring therefore was removed by substitution with alanine, and the model subjected to a round of refinement with simulated annealing in CNS [42]. Recalculation of phases revealed electron density extending from the backbone towards a new position for the Tyr80 side-chain. Positive difference electron density in m*F*_o _– *DF*_c _maps contoured at 3 sigma then revealed the location of the ligand in the RTA active site cleft. Amide-containing ligand molecules were built and energy minimized with Insight II, then positioned manually in the electron density at the active site. Manual model rebuilding was done using σ_A_-weighted 2m*F*_o _– *DF*_c _and m*F*_o _– *DF*_c _maps [43] in XtalView after successive rounds of crystallographic refinement with the ligand. Water molecules were added automatically to 3 sigma peaks in XtalView, and then evaluated individually. Sulfate molecules were added to strong tetrahedral peaks of electron density on the surface of the protein. The final refined model was evaluated using PROCHECK [44] and found to have good geometry. Molprobity [45] was used for model validation by an all-atom contact analysis and to guide optimization of side-chain rotamers. The coordinates and structure factors for RTA-NMU (2PJO), RTA-urea (2R2X), and RTA-acetamide (2R3D), and RTA-adenine (2P8N) have been deposited in the Protein Data Bank.

## Abbreviations

RTA: ricin A-chain, CD: circular dichroism, NMU: N-methylurea, H-bond: hydrogen bond

## Authors' contributions

All of the authors contributed to both data acquisition and analysis.
